# A rule-based theoretical account of social stories to address the double empathy problem

**DOI:** 10.3389/fpsyg.2023.1085355

**Published:** 2023-06-14

**Authors:** Louis John Camilleri, Katie Maras, Mark Brosnan

**Affiliations:** ^1^Centre for Applied Autism Research (CAAR), University of Bath, Bath, United Kingdom; ^2^Department for Inclusion & Access to Learning, University of Malta, Msida, Malta

**Keywords:** autism, Social Stories™, theoretical account, psychological mechanism, rule-based, systemizing, double empathy problem

## Abstract

Social Stories™ (SS) is one of the most popular and researched interventions for autistic children. To date, research that focuses on outcomes has been prioritized over the investigation of the psychological mechanisms that inform the intervention. In this article we consider theoretical accounts proposed thus far which could underpin SS. We argue that mechanisms that are based on social deficit theories lack validity, and propose a rule-based theoretical account to inform a strengths-based approach toward conceptualizing the mechanisms that underpin SS. We apply this account to the ‘double-empathy problem’ to propose that SS can be adapted to involve all parties in the development and delivery of SS support by adopting a rule-based perspective. We use the example of systemizing (the drive to analyze and explore systems in terms of ‘if-and-then’ rules), which is proposed to be a relative autistic strength, as a form of rule-based thinking that can provide a theoretical account of SS and a framework to address the double-empathy problem.

## Introduction

Social Stories™ (SS) (Gray and Garand, 1993; Gray, 1998) is one of the most frequently used story-based interventions (see Pane et al., 2015) by parents of autistic children^1^ (Green et al., 2006; Hess et al., 2008) and by practitioners who support autistic individuals (Smith and Gillon, 2004; Hsieh et al., 2018). Kokina and Kern’s (2010) analysis of SS literature indicates that SS research prior to 2010 focused on utilizing SS to support autistic individuals in a variety of tasks such as the reduction of inappropriate behaviors, improvement in social behaviors, supporting the acquisition of academic and functional skills, and assisting in novel events/transitions. The concern with reducing behaviors, and the labeling of autistic behaviors as ‘inappropriate’ by others (typically non-autistic others), could be indicative of a deficit outlook, as opposed to the neurodiversity-affirming perspective which is adopted in this article.

More recently, SS have also been used to support autistic children’s understanding in various contexts such as abduction prevention skills (e.g., Kurt and Kutlu, 2019), giving compliments (e.g., Almutlaq and Martella, 2018) turn-taking skills (e.g., Malmberg et al., 2015), greeting skills (e.g., Kagohara et al., 2013), and menstrual care (e.g., Klett and Turan, 2012). SS have also been used to support autistic children’s episodic memory (e.g., Hutchins and Prelock, 2018) and to increase task engagement (e.g., Cihak et al., 2012), amongst other purposes.

The SS intervention is described as a highly acceptable intervention by practitioners in the field of autism (Styles, 2011). Attitudes toward the intervention are also reported to be positive (Dodd et al., 2008; Acar et al., 2017; Camilleri et al., 2022). The low cost of the intervention and its availability are possible factors that contribute to the positive attitudes toward the intervention amongst practitioners and parents. Whilst SS outcomes research is extensive, a great degree of variability in terms of outcomes is reported in the SS literature (Camilleri et al., 2021). The literature also highlights a lack of a clear theoretical underpinning or rationale for SS (Reynhout and Carter, 2006; Camilleri et al., 2021). However, it is not uncommon for psychological studies to focus on measuring outcomes, especially since this would inform the very pertinent question of whether an intervention is effective (Windgassen et al., 2016).

The recent increase in interest in the use of digital technology as a means to develop and deliver SS (e.g., Constantin et al., 2013, 2017; Hanrahan et al., 2020; Smith et al., 2020, 2021; Camilleri et al., 2021; Saeed and Safi, 2020; Safi et al., 2022) has been accompanied by a further interest in understanding the intervention’s mechanisms and theoretical underpinnings (e.g., Riga et al., 2021). The investigation of mechanisms underpinning SS can contribute to two very important aspects of psychological interventions or supports. First, it advances our understanding of the key psychological processes, or mechanisms (i.e., the processes involved in, or responsible for, an action to occur), that affect positive outcomes of SS. Second, it also helps to locate issues and questions within existing theories and conceptualization of the SS support tool. In turn, the investigation of a possible theoretical rationale, or mechanism, may assist in the evaluation, optimization, and development of further approaches or support tools (Bawazir and Jones, 2017).

Thus, to continue to build on the limited, but developing, discussion on the underpinning mechanisms that inform the SS intervention, in the first part of this article we consider the theoretical accounts for SS which have been proposed thus far. Subsequently, in the second part, we propose a ‘rule-based’ theoretical rationale for SS. This rule-based rationale is informed by a neurodiversity paradigm that defines autism as “a natural identity with strengths and weaknesses” (Gillespie-Lynch et al., 2017, p. 3). We also employ the ‘systemizing’ theory (Baron-Cohen and Lombardo, 2017) as an example of rule-based thinking to inform our discussion.

Autism is clinically defined in terms of difficulties in social communication and interaction and patterns of restricted and repetitive behaviors, interests and activities (American Psychiatric Association, DSM-5 Task Force, 2013; World Health Organization, 2004). However, researchers and self-advocates have called for more strengths-based approaches to our understanding of autism (see Urbanowicz et al., 2019), incorporating strengths such as the ability to hyperfocus, attention to detail, good semantic memory, and deliberative analytical thinking (e.g., Brosnan et al., 2016, 2017; Russell et al., 2019; Urbanowicz et al., 2019). Such strengths are considered “context-dependent,” and are not necessarily fixed traits (Russell et al., 2019). Nevertheless, this strengths-based lens/framework is contrasted with social deficit-driven epistemology, which is argued to stem from the medicalization of the autistic individual (Kapp, 2019).

In this article we utilize a neurodiversity paradigm (Singer, 2016) which views autism as a natural and valuable form of human diversity (Chapman, 2020). This neurodiversity paradigm, as described by Dwyer (2022), challenges the view that communication difficulties, or disability, exist within the autistic person alone. Rather, disability is the result of the interaction between the characteristics of an individual and the environment around that person. Similarly, in this article, we utilize what Milton refers to as the double empathy problem (Milton, 2012) to challenge social deficit-driven ontologies, and propose that SS can contribute toward addressing the double-empathy problem by involving all parties in the SS process by adopting a rule-based perspective, which could be considered an autistic strength.

## Social Stories™

Social Stories™ (SS) were introduced 30 years ago by Gray and Garand (1993), and are short narratives that follow specific guidelines to objectively describe a person, skill, event, concept, or social situation (Gray, 1998; Timmins, 2016). They are personalized and detailed accounts written specifically to provide a narrative of a situation (refer to Box 1 for an example of a SS). SS sometimes also can include visual aids which accompany the narratives, such as line drawings, photographs or cartoons. These aids are aimed toward illustrating or highlighting the key points of the story.

Social Stories™ aim to facilitate the accurate transfer of information between an author (i.e., the person developing the story) and the individual that the story is written for; also known as the audience (Gray, 2010). Each story that is developed is tailored to the individual’s abilities, attention span, and learning style and consists of three parts: introduction, body, and conclusion (Howley and Arnold, 2005). The stories are written in first- and/or third-person perspectives and have a positive and patient tone. Stories should inform and answer ‘where,’ ‘when,’ ‘who,’ ‘what,’ ‘how,’ and ‘why’ questions, and consist of more descriptive sentences than coaching sentences. Descriptive sentences are sentences that describe the facts relating to the situation clearly and objectively, whilst coaching sentences are sentences that describe or suggest responses or actions. Social stories can also include perspective sentences and affirmative sentences. Perspective sentences describe the internal states, feelings, and beliefs of other people, whilst affirmative sentences tend to highlight or stress an important point. The social stories should then be developed and refined in a way that should help to maximize the transfer of information (Gray, 2010).

BOX 1 **Example of a social story.**

**Table 1:** 

Title of the story: Giving a gift A gift is something special that one person gives another person. A gift can be a toy, an object, or an accessory. When it is a birthday, a special occasion, or a holiday, people give gifts to one another. A gift is given to celebrate a birthday or holiday. A gift is also given to make people happy. People enjoy giving gifts. People also enjoy receiving gifts. If I give a gift to my friend, my friend will become the owner of the gift. A gift is something special that one person gives to another person to keep.

The social story in Box 1 has been taken from the Stories Online for Autism (SOFA, sofa-app.org) digital application.

Over time the guidelines for the development of SS, as defined by Gray and Garand (1993) have been updated. Gray (2022) states that these changes can be seen more as revisions and reorganizations which resulted from research and experience of using SS. The latest revision of these guidelines (Social Stories 10.3) was introduced by Gray in 2021 with 10 criteria for the development of SS. These guidelines emphasize the need for stories to (1) humbly, yet accurately, share social information with the audience, (2) be informed by in-depth understanding of the audience, (3) have one title, a three-part structure (introduction, body and conclusion), and descriptive and coaching sentences, (4) be tailored to the abilities, attention span, learning style and, whenever possible, talents and interests of the audience, (5) have a patient and supportive ‘voice,’ and (6) answer relevant ‘who,’ ‘what,’ ‘when,’ ‘where,’ why and ‘how’ questions. They also specify for stories to (7) celebrate and praise, and (8) consist of three times as many descriptive sentences as coaching sentences. Criteria 9 and 10 highlight the iterative process of refining and improving the story in order to ensure that Social Humility and the 10 criteria that guide social story development are consistent with its introduction and review over time (Gray, 2021). Finally, the 10 criteria must be adhered to for a story to be considered a Social Story™ (Wright et al., 2016).

BOX 3 **Example of a social story, focusing on sharing information about novel events, analyzed in terms of if-and-then patterns.**

**Table 3:** 

Social story text	Analysis in terms of if-and-then patterns	
I’m a child, and I am growing taller and bigger.	IF (INPUT): People grow	Causal Pattern 1
All children grow.		
Their clothes stay the same size.	AND (OPERATION): Clothes remain the same size/clothes do not grow	
For this reason, children’s clothing fits for a few months or so.	THEN (OUTPUT): Clothes will not fit	
The time comes when clothing is too small.	IF (INPUT): Clothes are small	Causal Pattern 2
Shoes may fit tight and toes may feel crowded inside shoes.	AND (OPERATION): Clothes are tight when I try them on.	
Or, pants are tight or short.		
Sometimes, shirts get hard to button.		
It’s time for new clothes.	THEN (OUTPUT): Time for new clothes	
I need new clothes because I get bigger, and my clothes stay the same size.	SUMMARY	

The social story in Box 3 has been taken from The New Social Story Book, by C. Gray, p. 35. Copyright 2010 Carol Gray.

Gray and Garand (1993) and Gray (2022) states that these guidelines are based on a number of overarching principles. The first, according to Gray (1998, p. 168) is the need to “abandon all assumptions.” Gray (1998) argues that what makes it difficult for autistic and non-autistic people to understand each other and interact is that mutual social understandings are based on assumptions that could be erroneous or inaccurate. Thus, Gray’s second principle states that there must be a recognition that social impairment in autism is a result of erroneous assumptions made on both sides of the social equation. Accordingly, whilst acknowledging possible inaccuracies and imperfections in all assumptions related to social interactions, Gray (2018) states in her third principle that each interaction, and each person’s idiosyncratic perspective, is equally valid and deserving of respect. The most recent version of the guidelines includes more focus on what Gray (2022) describes as ‘social humility’. This concept is meant to encourage the acknowledgement of authors’ inherently fallible assumptions related to social interaction. Thus, social humility aims to decrease the chance of the authors’ erroneous understanding of individuals identified as autistic, through embracing the perspective of the autistic individual.

BOX 4 **An example of a social story, focusing on sharing information about a greeting, analyzed in terms of if-and-then patterns.**

**Table 4:** 

Social story text	Analysis in terms of if-and-then patterns	
There are many ways to greet someone.	Introduction	
When I see someone I know, especially if I am seeing that person for the first time that day, it is friendly to say “hello.”	IF (INPUT): I see someone I know And (OPERATION): I am seeing that person for the first time that day. Then (OUTPUT): I say hello	Causal Pattern 1
They may say “hello” too. They may stop to talk with me.	IF (INPUT): If I say hello And (OPERATION): They say hello too Then (OUTPUT): They may stop to talk with me.	Causal Pattern 2
Sometimes people shake hands to say “hello.”	IF (INPUT): If I say hello And (OPERATION): They shake hands Then (OUTPUT): That means hello	Causal Pattern 3
People may try to shake my hand if they are meeting me for the first time.	IF (INPUT): People try to shake my hand. And (OPERATION): People are meeting for the first time. Then (OUTPUT): That means hello	Causal Pattern 4
This will happen more and more as I get older.	Comment on the frequency of the causal pattern by introducing another causal pattern: IF (INPUT): I am getting older And (OPERATION): People meet me for the first time Then (OUTPUT): I will shake more people’s hand.	Causal Pattern 5
Once in a while, I go to visit relatives or close friends. A short hug as I arrive means hello.	IF (INPUT): I go to visit relatives or close friends. And (OPERATION): I receive a hug Then (OUTPUT): That means hello	Causal Pattern 6
Sometimes, if I am just passing someone I know, I may smile, wave, or just nod my head.	IF (INPUT): I am passing someone. And (OPERATION): I smile, wave or nod. Then (OUTPUT): That means hello.	Causal Pattern 7
If I said hello to that person earlier in the day, smiling, waving, or nodding my head means “Hello again.” This is a friendly thing to do.	IF (INPUT): I am seeing a person I saw earlier. And (OPERATION): Nod my head. Then (OUTPUT): That means hello again.	Causal Pattern 8

The social story in Box 4 has been taken from The New Social Story Book, by C. Gray, p. 83. Copyright 2010 Carol Gray.

### Original theoretical rationale for social stories: social-deficit perspectives

Gray (1998) had proposed that the rationale for SS is based on what at the time represented the growing understanding of social cognition in autism. She argues that Theory of Mind (ToM, Baron-Cohen et al., 1985; Leslie, 1987; Happé and Frith, 1995), as well as Weak Central Coherence (WCC, Frith, 1989; Happé, 2000; Happé and Frith, 2006), are important areas of research to SS. ToM is defined as the capacity for prediction and interpretation of behavior by using representations of hidden causally efficacious mental states (Westra and Carruthers, 2018); that is, the ability to attribute beliefs, emotions, intentions, and goals to ourselves and others. Central Coherence is defined as the cognitive tendency to process information comprehensively, globally, and in context (Noens and Van Berckelaer-Onnes, 2008). Adequate central coherence aids individuals in making sense of the ‘whole picture’ and seeing structure and meaning. Baron-Cohen et al. (1985) describe how autistic individuals could have difficulties in ToM, whilst Happé and Frith (2006) describe how autistic individuals could present with weak central coherence (i.e., with a preference for processing local detail over global processing).

Using ToM and WCC theories as a foundation, Gray (1998) argues that SS provide an autistic individual with access to a social “secret-code” (p.169) and thus compensate for that individual’s poor mind-reading, or mentalizing (ToM), abilities. Gray (1998) states that SS also “raise awareness of yet another secret” (Gray, 1998, p. 169) by integrating information at different levels, and thus provide a more meaningful and contextual (central coherence) understanding of everyday activities. Tassini et al. (2021) suggest that the drive for local processing, and the ensuing tendency to focus on a specific part of a situation, may lead to difficulties in understanding the bigger picture.

Thus, through the WCC lens, SS could be seen as providing explicit information that contributes toward a more meaningful understanding of everyday activities. However, if there is a bias, or strength, toward local processing (see Koldewyn et al., 2013; Scher Lisa and Shyman, 2019), it is unclear how SS can help audiences with overcoming that bias and understand the context from a more global perspective. Thus, whilst it is possible that SS could be used to highlight global aspects of a situation (such as the context), there is no empirical evidence exploring a link between WCC and SS and such a rationale has been criticized for having “no real theoretical engagement” (Bawazir and Jones, 2017, p. 533).

Gray’s first and second versions (10 and 10.1, Gray, 2015) of her criteria specifically advise on the inclusion of perspective sentences that are aimed to make explicit the perspective of others. The impact of perspective sentences in SS, specifically on improving the audience’s adaptive behaviors, was investigated by Okada et al. (2008) who found the inclusion of perspective sentences had little to no impact on improving target behaviors. Thus, the use or inclusion of perspective sentences is not explicitly detailed in Gray’s 10.2 (Gray, 2018) and 10.3 (Gray, 2021) guidelines. Rather, the 10.3 guidelines (Gray, 2021) emphasize further the need for the authors to recognize the uniqueness of every human experience and perspective. Perspective-taking is alluded to, through the term ‘social humility,’ and aims to ensure that the authors, rather than the audience, make a concerted effort to see to potential difficulties related to misunderstandings in perception. Thus, there seems to have been an increase in emphasis on the concept of mutual misunderstanding. This is reflected in the evolution of the SS criteria. Initially, Gray and Garand (1993) and Gray (2015) urged authors to “improve their understanding of the audience” (Criteria 10.1). In the latest criteria, the term “Social Humility” is included specifically in the first criterion (10.3. Gray, 2021). Thus, whilst the notion of acknowledging mutual misunderstandings of both the author and audience of the SS has always informed Gray’s (1998) philosophy, in a later version of her criteria (10.3) the term “Social Humility” takes center stage.

The heightened focus and explicit use of the term “Social Humility” in SS criteria is also suggestive of the shift in the ontological account of autism literature; from a view of a social communication deficit underpinned by poor ToM (medical model) to a social relational (see Thomas, 2004) view of a shared misunderstanding of social communication (between autistic and non-autistic communication partners). Specifically, the (medical model) view that an autistic person’s inability to read the subtext of a social situation is the central deficit in autism has been challenged by Milton (2012, 2014, 2017), who argues that ToM (also termed cognitive empathy; see Mazza et al., 2014), is bi-directional. Milton states that whilst autistic people may present with a particular way of experiencing the world, this alone does not explain the breakdown in reciprocity and mutual understanding between autistic and non-autistic individuals. Milton (2012) proposes the ‘double empathy problem’, whereby poor mutual understanding is not due to autistic cognition alone (see Crompton et al., 2020). Rather, it is resulting from difficulties with reading each other’s minds. That is, autistic and non-autistic individuals who are interacting both have difficulties ‘reading’ their autistic or non-autistic counterparts. Milton (2017) refers to this phenomenon as a mismatch of salience. Hence, it is argued that “the problem is just as much one for the non-autistic person as for the person with autism” (Chown, 2014, p. 1672). This view, that interaction takes two, was investigated further by Brewer et al. (2016), Edey et al. (2016), and also by Heasman and Gillespie (2018), who indicated that mental states and expressions of autistic people were poorly recognized by non-autistic individuals.

### Social learning and constructivist explanations

Bawazir and Jones (2017) attempt to explain the mechanisms behind SS by using Bandura’s (1971) social learning theory, in terms of learning experiences that involve observation, modeling, reinforcement, and the consequent cognitions that emerge from these learning experiences. Bawazir and Jones (2017) argue that the development and delivery of SS activate the components necessary for observational learning to occur (i.e., maintaining attention, retaining information, motoric reproduction, and motivation). Thus, they maintain that the changes in the responses of autistic individuals, as a result of using SS, could be explained by using social learning theory.

More recently, Riga et al. (2021) have suggested that social stories rely on Social Constructivism theory. Social Constructivism is a theory proposed by Vygotsky (1968) which suggests that knowledge is socially constructed through interaction with others and the use of socio-cultural tools. From a Social Constructivist perspective, a SS could be considered a socio-cultural tool that provides opportunities for creating shared meaning. Riga et al. (2021) argue that SS create opportunities for meaningful interaction between the author and the audience, and thus help in constructing shared understanding.

Social constructionism and social learning theory emphasize two different aspects of learning. The former refers to the construction of knowledge through collaboration whilst the latter the acquisition of new behaviors through modeling and imitation. Interestingly, both theories advocate for the importance of interactive and social elements in the learning or transmission of knowledge. Thus, in utilizing such theoretical frameworks, both Bawazir and Jones (2017) and Riga et al. (2021) allude to the importance of active engagement between the author and the audience of the SS intervention.

### Further proposed theoretical explanations

Rowe (1999) utilizes the Piagetian notion of schemas (or schemata) to explain the SS mechanism. Schemas are mental units of understanding. They are representations of experiences that can be organized into complex relationships with one another, which then contribute toward a person’s view of the world. According to Rowe (1999), SS can help the audience to scaffold a schema which they have not developed yet. Or it can help to make an implicit schema explicit. Thus, by sharing factual information about an event or experience (i.e., by scaffolding a schema), a SS can help increase an autistic person’s understanding of a situation.

As SS have been used to retell an event that has occurred in the autistic child’s life, and also to link an individual’s experience to their future planning, the role of episodic memory has been highlighted by Hutchins and Prelock (2018). The authors suggest that SS can support a rich personal recall of an event to ensure a greater impact of the intervention.

Reynhout and Carter (2011) provide the only review of the theoretical rationales underpinning SS to date. Besides proposing ToM and WCC (also referred to as ‘strong specific coherence’) theories, Reynhout and Carter (2011) also propose executive functioning, stimulus over-selectivity (also referred to as ‘strong stimulus selectivity’), visual learning style, and language comprehension as perceptual and cognitive characteristics that may shed light on potential mechanisms underpinning SS. Specifically, they suggest that SS may support executive functions (which are mental control processes such as inhibition, working memory, cognitive flexibility, and planning) in autistic individuals and as a result, support planning and organization. Reynhout and Carter also suggest that autistic individuals may have a tendency for “strong stimulus selectivity.” This is the tendency to attend to one specific aspect of a situation or stimulus rather than seeing the “whole field” (Reynhout and Carter, 2011, p. 373). Thus, according to the authors, SS can make aspects of the situation which are necessary for a complete understanding of the situation more explicit.

A visual learning style (or preference) in autism has also been proposed by Reynhout and Carter (2011) to explain how visually cued instruction and written language in SS could serve as instructional supports. Furthermore, the explicit direction given, in terms of ‘who, what, when, where and why,’ may also contribute toward compensation for difficulties in language comprehension by making important information more prominent.

Finally, Reynhout and Carter (2011) discuss a behavioral explanation which is informed by antecedents and consequences. This behavioral explanation proposes that SS develop “loose contingency contracts” (p. 375). The authors argue that SS make antecedents and consequences of behaviors explicit. In other words, SS could be explained in terms of rules and patterns, where SS provide clear links between behaviors and their subsequent outcomes: i.e., if you do this activity, then you will get this outcome. Reynhout and Carter (2011) also draw on Demiri’s (2004) hypothesis on how SS rely on the use of rule-governed behavior to learn to respond appropriately in a given situation without having previous experience of dealing with similar contingencies in the past. From this perspective, SS highlight the appropriate antecedent events that signal that a particular behavior is necessary. The effectiveness of such contingencies approach is explored by Bradley and Noell (2021) who investigated the effectiveness of an intervention program designed to promote the acquisition and generalization of rule-governed social skills in autistic children. They argue that “rule-governed behavior allows individuals to behave according to contingencies that may not be explicitly stated or that have never been contacted directly” (Bradley and Noell, 2021, p. 2). Social stories, from this perspective, also indicate how the audience is expected to respond whilst also specifying the consequences or outcomes that will occur as a result of the audience’s response. This, according to Reynhout and Carter (2011) “would seem to be an attractively parsimonious explanation” (p. 376).

### Novel theoretical rationale for social stories: rule-based perspectives

Taken together, there are two elements to the proposed theoretical accounts for SS thus far which have been highlighted repeatedly: (1) make implicit social information explicit, and (2) actively involve both communication partners. To inform further the SS mechanism discussion, strengths-based perspectives should be seriously considered. A strengths-based approach to understanding autism emphasizes strengths, or preference, in explicit deliberative analytical thinking. Thus, a theoretical account of SS that embraces collaborative and explicit perspectives as well as emphasizes rule-based thinking may be useful within the SS mechanisms debate, and also for addressing the double empathy problem.

A strength in rule-governed processing has been proposed to characterize autism. Theoretical accounts of deliberative analytical thinking strengths in autism, such as the Dual Process Theory of Autism (Brosnan et al., 2016, 2017; Lewton et al., 2019; Ashwin and Brosnan, 2020; Brosnan and Ashwin, 2022a,b), may also be a potential framework for understanding the mechanisms underpinning social stories. The Dual Process Theory of Autism proposes an autistic preference for slower, explicit, deliberative, analytical processing (compared with a non-autistic preference for rapid, implicit, intuitive processing). Deliberative analytical processing incorporates the mental manipulation of cause-effect relationships (Crespi, 2021). A specific form of processing cause-effect relationships has been termed ‘systemizing’, which is the drive to construct rule-based systems that function in an ‘if-and-then’ manner. When systemizing, the input is ‘IF,’ the operation is ‘AND,’ and the output becomes ‘THEN’ (Baron-Cohen and Lombardo, 2017). Systemizing is therefore a specific example of a rule-based perspective that may be useful for understanding the mechanisms underpinning SS.

### What is systemizing?

Systemizing is a drive to (1) analyze the variables in a system, (2) derive the underlying rules that govern the behavior of a system, and (3) construct systems that allow for prediction to occur (Baron-Cohen et al., 2003, 2009; Baron-Cohen, 2021). Here, a system is defined as something that takes inputs, which can then be operated in variable ways, to deliver different outputs in a rule-governed way. Baron-Cohen et al. (2003) propose at least six kinds of systemizing domains – Technical, Natural, Abstract, Social, Organizable, and Motoric – which share this same underlying if-and-then process of pattern-seeking.

Baron-Cohen and Lombardo (2017) propose a ‘systemizing mechanism’ that undertakes the three key cognitive processes of input (IF), operation (AND), output (THEN). They also speculate on the neural basis of systemizing. Rather than one discrete neural ‘module,’ they propose a number of neurological processes which could explain the higher-than-average rule-seeking drive of autistic individuals. These are: a heightened initial low-level processing of sensory/perceptual input, a heavily biased attentional process that promotes a detail-oriented focus, and a motivational component that impacts an individual’s drive to understand systems, involving the brain’s reward system through dopaminergic neurons.

Systemizing is common amongst all humans (Wakabayashi et al., 2007; Kidron et al., 2018; Naor-Ziv et al., 2021; Van Der Zee and Derksen, 2021). Evidence suggests that autistic individuals have higher levels of systemizing than non-autistic individuals (Baron-Cohen, 2006). An intense search for structure is termed ‘hyper-systemizing’ (Baron-Cohen et al., 2009; Van Der Zee and Derksen, 2017). Hyper-systemizing also refers to a tendency to be change-resistant whilst also presenting with a cognitive style that is apt at law-based pattern recognition (Baron-Cohen and Lombardo, 2017). The hyper-systemizing process can be informed by greater attention to detail (Baron-Cohen et al., 2009; Brosnan et al., 2012). Rutherford and Subiaul’s (2016) study, which focuses on the motivational urge for systemizing, indicated that autistic children showed a stronger explanatory drive than non-autistic children. The authors use the term “explanatory drive” to explain an individual’s desire to explain ambiguity and to explain how systems work.

The evidence for hyper–systemizing being characteristic in autistic individuals is increasing, as established in Van Der Zee and Derksen’s (2021) narrative literature search, which provides a comprehensive overview of the state of research on systemizing. The authors also suggest that measuring an individual’s systemizing abilities could be useful in distinguishing between autistic individuals and non-autistic individuals (also see Baron-Cohen et al., 2003; Wheelwright et al., 2006; Auyeung et al., 2009).

## Discussion

### Systemizing and social stories

Many everyday experiences can be described in terms of input-operation-output systems that can be lawfully explained. The examples in Box 2 are potentially causal patterns that can be inferred in terms of a rule or law. Each of these examples pertains to a different class of systems; the first is the natural system, the second can be considered a social system, whilst the third is a numeric system. In all three, cases, when all three elements line up, the system repeats itself and a causal pattern is observed (Baron-Cohen and Lombardo, 2017).

BOX 2 **Example of if-and-then systems.**

**Table 2:** 

IF [input]	AND [operation]	THEN [output]
An apple is unsupported	There is a gravitational force	It will fall toward the earth
I receive a present	I want to be polite	I must say thank you
I leave home at 3 pm	And walk at a brisk pace	I will arrive at the station at 330 pm.

Such If-And-Then patterns can be found in examples of SS. This can be seen in the SS presented in Box 3. In this SS, the goal is to share information about a novel event in preparation for when new clothes will be bought. The SS in Box 3 consists of two causal patterns; one building on the other. Another example of causal patterns in SS is in Box 4, which consists of eight causal patterns. This story focuses on supporting understanding within a social domain by sharing information on ‘greeting someone.’ Neither story is written using strict if-and-then vocabulary. Rather, they are stories that meet Gray’s SS criteria in that they are positively written, have a patient tone, are guiding and not directing, and are balanced in terms of having an introduction, body and conclusion. The analysis described in Box 4, which was carried out *post hoc*, highlights the causal patterns (in input-operation-output format) in the story. It evidences a number of “qualifiers” such as “if,” “may,” “try,” “more,” and “or.” Such words contribute to a more authentic description of the theme in question, and are used to communicate qualities of the causal patterns; such as the frequency of the pattern, as well as different iterations of the pattern. Therefore, the analyses in Boxes 3, 4 highlight how SS may be utilizing causal patterns, to engage more effectively the audience’s exploratory drive.

Thus, by following Gray’s Criteria, particularly in relation to (1) ensuring that SS answer ‘where,’ ‘when,’ ‘who,’ ‘what,’ ‘how’ and ‘why’ questions, and (2) focusing on describing over directing through more descriptive than coaching sentences (Gray, 2010, 2015), SS can offer more opportunities for highlighting the if-and-then (input-operation-output) framework described by Baron-Cohen and Lombardo (2017) whilst emphasizing knowledge and mental processes. As a result, it can be argued that SS can (1) offer opportunities for more efficient systemizing by providing the necessary information for the if-and-then process to ensue, and (2) offer opportunities for systemizing through observation (i.e., by observing an operation unfolding in the story, as illustrated in Box 2).

Recent SS research suggests that SS can be useful for increasing understanding (as Gray originally suggested) whilst also reducing anxiety in autistic children (Smith et al., 2020, 2021). This increase in understanding is consistent with the notion of how rule-based strengths of autistic individuals (e.g., systemizing) can be underpinning the SS mechanism, as SS are a means to communicate and identify rules and causal patterns. Increased understanding may therefore serve to also increase a sense of certainty in predicting future events as described by the SS. Autism is associated with elevated levels of intolerance of uncertainty, which mediates between autistic traits and anxiety (Boulter et al., 2014; Vasa et al., 2018; Jenkinson et al., 2020). It has recently been suggested that social situations are difficult for autistic individuals precisely because of their inherently uncertain nature (rather than their social nature *per se*, Berkay and Jenkins, 2022).

Thus, SS can help to predict patterns and reduce uncertainty. However, due to their dynamic, complex, and uncertain nature, some social communicative acts (such as detecting the ironic intent of a speaker or negotiating eye contact) may not be predictable. In fact, there is no evidence (see Kokina and Kern, 2010; Camilleri et al., 2021) that suggests that SSs could be used successfully in contexts which are so dynamic. Similarly, Baron-Cohen (2002, p. 248) also states that “systemizing is of almost no use when it comes to predicting moment-by-moment changes in a person’s behavior.” Nevertheless, SSs could still be potentially used to target social communicative goals which are less dynamic. They could also be used to target aspects (or elements) of these processes which could be more predictable. Furthermore, it is unlikely that one story alone can explain the complexity of a social communicative act. As highlighted in Gray’s criteria (see Gray, 2021), ideally a number of complementary social stories should be used (Criterion 10: Mix and Match to Build Concepts) to help develop further the understanding of complex and dynamic concepts.

Hence, it is possible that systemizing is a mechanism underpinning SS, as the SS encourages authors to explicitly share information that is informed by the audience’s propensity for systemizing. This may, in turn, increase certainty in the autistic individual with respect to the content of the SS which reduces their level of anxiety (as an elevated intolerance of uncertainty relates to greater anxiety). In this way, SS can be seen as a mechanism of support for autism that builds upon autistic strengths in systemizing, rather than addressing weaknesses in ToM (or cognitive empathy). If the author constructs the SS with the rule-based communication preferences of their audience in mind, a systemizing account of SS embraces Gray’s principle of ‘social humility’ to address Milton’s ‘alignment of salience’ that characterizes the double empathy problem.

### Social stories and the double empathy problem

The original definition of SS given by Gray and Garand (1993) highlights the process of sharing information between audiences and authors. By recognizing that in using a SS as a tool to communicate accurate information, there is also the recognition that there are two people involved in the exchange. Gray (1998) argues that it is important for authors to acknowledge that the perspectives of non-autistic people and the perspectives of autistic people could be different. The social stories that are developed, usually by parents or practitioners, are developed by first gathering information that helps improve the authors’ understanding of the audience in relation to the goal to be reached. This first informs the author who is trying to understand the current situation from the audience’s perspective (i.e., trying to walk in the autistic person’s shoes). Gray (2021) recommends that authors engage with the audience to gather their thoughts, feelings, and perspectives, and subsequently use this knowledge to inform the stories. This process, as described by Gray (2021), can be the process with which the double empathy problem, as described by Milton, is addressed. In this manner, the story development process helps create a platform from which the audience’s (i.e., the autistic person) perspective is better understood. This joint effort can be completed through verbal or nonverbal interchanges, and should focus on using various strategies for engagement and the sharing of information between non-autistic and autistic individuals. Structuring this joint effort in an explicit, logical and systematic way can help to mediate between the non-autistic and the autistic individual’s communication preferences, and in doing so potentially reducing what Milton (2012, 2017) refers to as a mismatch of salience between the autistic and non-autistic individual.

SS are typically written by non-autistic authors, for autistic audiences. If systemizing tends to be higher in autistic individuals compared to non-autistic individuals, it may be that non-autistic authors need to adjust more than the autistic audience to present SS in an if-and-then format. This would enable SS to support relative autistic strengths in systemizing. If-and-then patterns can enable autistic individuals, as well as non-autistic individuals, to analyze objects or events in terms of small units and enable focus on one detail (i.e., the input) at a time. Furthermore, the SS provides clear information on how the input is transformed, operationalized, or manipulated (i.e., the operation). Finally, the SS will also highlight the outcome of the operation on the input (i.e., the output). In this manner, a SS could be seen to create a meaningful and accurate if-and-then pattern through which both the author and the audience may infer and operationalize a rule about the world around them. In this way, the SS process (i.e., the development and delivery of the SS) could be harnessing the autistic individual’s strong explanatory drive (Rutherford and Subiaul, 2016), the autistic individual’s preference for structured patterns (Strathearn et al., 2018), and the autistic individual’s capacity for systemizing (Van Der Zee and Derksen, 2021).

### Future research

We recommend that future SS research emphasizes investigating two aspects highlighted in this article: (1) if-and-then patterns in social stories, and (2) social humility in the development (or co-development) of social stories. SS research can include analysis of stories in terms of if-and-then patterns or systems to ascertain if social stories that highlight such patterns are more likely to result in positive outcomes when compared to social stories which do not highlight such patterns.

As highlighted in this article, the function of systemizing is to identify laws, rules and/or regularities that govern a system in order to understand how that system works and to predict what it will do (Baron-Cohen and Lombardo, 2017). However, accurate prediction is not always possible, especially because some everyday situations (particularly social situations) contain high levels of uncertainty. However, SS (as described in Box 4) can comprise causal hypotheses, in the form of classic logic (i.e., that each proposition has a truth value of either “true” or “false,” but not both; see Pijnacker et al., 2009) whilst also hypothesizing scenarios with “extra information” (i.e., information that could invalidate or challenge the original causal relationship). Future research could focus on investigating if SS with multiple causal patterns, compared to SS with little to no causal patterns, can support further understanding of complex and dynamic everyday social situations and if they can help mitigate the exceptions encountered in real-world social situations.

The extent to which perceived uncertainty is a mediating factor addressed by SS can also be explored. Many aspects of social communication and interaction do not have lawful regularities that are amenable to identifying a single input to operate on, and are inherently uncertain (Berkay and Jenkins, 2022). SS may reduce uncertainty in a situation, however, Baron-Cohen (2002) states how systemizing is not effective in anticipating the fluctuations in someone’s “moment-to-moment” (p. 248) behavior. Speculatively, this may relate to SS being more effective for preparation for novel and predictable events, than for facilitating communication (e.g., Hutchins and Prelock, 2013). Golan and Baron-Cohen (2006) found that whilst emotion recognition could be developed through systemizing, there was limited broader generalization beyond what was explicitly learned. Thus, it may be that SS effectiveness is related to lawfulness of the situation, and may have limited generalization. This may be a way for informing when SS are, and are not, likely to be effective.

Furthermore, if systemizing is a drive as well as a cognitive process (Baron-Cohen and Lombardo, 2017), and a strength in rule-based systemizing is underpinning the SS support tool, individuals who are apt systemizers should benefit more from utilizing SS. Thus, measures such as the Adolescent Systemizing Spectrum Quotient (Auyeung et al., 2012) could be utilized to investigate if high systemizing scores can predict more positive SS research outcomes. Other measures for the identification of rule-based strengths, such as Pacini and Epstein’s (1999) Rational Experiential Inventory (as used in Dual Process Theories, see Brosnan et al., 2016) could also be utilized to investigate the relationship between possible aptitudes for rule-based deliberative analytical thinking styles and SS effectiveness. Future research can explore if systemizing relates to effectiveness in some categories of SS relative to other categories of SS (e.g., support learning a new skill vs. assisting in novel events/transitions) or if deliberative analytical thinking generally relates to SS effectiveness, rather than systemizing specifically.

The importance of social humility for the development of SS should also be investigated further. Importantly, the author may have to adapt to the style of the audience. This paper argues that SS can reconcile the diverse style of communication of autistic and non-autistic individuals and thus mediate the double-empathy problem. Thus, it is recommended for research to investigate if SS which have been mutually developed or co-constructed, to various degrees, together by autistic and non-autistic authors are more effective than stories that are developed solely by non-autistic authors. Autistic individuals self-delivering SS that they have self-developed (for example using the co-developed SOFA-app.org) also represents a potentially fascinating avenue of future neuroaffirmative research.

## Conclusion

This article argues that the investigation of outcomes of SS has taken priority over the investigation of the psychological processes underpinning the intervention. However, the identification of these mechanisms should be given more importance as it could locate more effectively issues and questions related to the effectiveness of the support tool, whilst contributing further to the issue of variability in outcomes research. In light of this, the article sheds light on some of the strengths and limitations of the theoretical mechanisms that have been proposed so far, and argues against those that are based on social-deficit ontologies of autism, as they are lacking a practical, philosophical and empirical foundation.

This article takes a neuroaffirmative approach toward the use of the SS support tool and SS research. This approach challenges the use of SS as a means to “reduce inappropriate behaviors.” The labeling of autistic behaviors as inappropriate by others (typically non-autistic others) that require reduction can be seen as stemming from a deficit model which implies that disability is a result of within-person factors which can be changed. In this article, we argue that disability is a result of the interaction of the person with the environment. From this perspective, SS could be used to reduce behaviors that autistic individuals themselves would like to reduce or support autistic individuals to interact with their environment. Consequently, we suggest that SS be used as Carol Gray originally intended, as a means to share accurate information effectively between author and audience.

This article reviews the growing evidence of the tendency for autistic individuals to prioritize rule-based thinking as a way of better understanding the world. This contributes to a strengths-based account of SS. Importantly, this article does not propose changing how SS are written, nor moving away from Gray’s 10 criteria. Rather, it propositions that (1) rule-based communication preferences of the audience are kept in mind when developing stories, and that (2) causal if-and-then patterns are used to analyze further the SS.

Finally, whilst the underlying mechanisms of SS are still speculative, and also, not necessarily mutually exclusive (i.e., more than one process may inform SS); at this stage, we propose that SS can encourage both the author and the audience to jointly analyze experience and events in terms of if-and-then patterns. Thus, a SS can provide the opportunity to address the double-empathy problem by highlighting a common if-and-then ruled-based structure to develop a shared understanding. Thus, the SS tool can serve as a support between non-autistic and autistic people. This is an efficient and parsimonious account of SS, which warrants further investigation.

## Data availability statement

The original contributions presented in the study are included in the article/supplementary material, further inquiries can be directed to the corresponding author.

## Ethics statement

All procedures performed in this study were in accordance with the ethical standards of the institutional and/or national research committee.

## Author contributions

All authors contributed to the conceptualization of the study. LJC wrote the first draft of the manuscript. All authors made a substantial, direct, and intellectual contribution to the work and approved it for publication.

## Conflict of interest

The authors declare that the research was conducted in the absence of any commercial or financial relationships that could be construed as a potential conflict of interest.

## Publisher’s note

All claims expressed in this article are solely those of the authors and do not necessarily represent those of their affiliated organizations, or those of the publisher, the editors and the reviewers. Any product that may be evaluated in this article, or claim that may be made by its manufacturer, is not guaranteed or endorsed by the publisher.

## Footnotes

^1^The way autism is, or should be, described is lacking consensus (Botha et al., 2021). However, autistic adults (Taboas et al., 2023) and autistic advocates (Sinclair, 2013) prefer identify-first language (i.e., autistic person/individual), over person-first language (i.e., person/individual with autism). Thus, in this paper identity-first language is used.
